# Accuracy of the interferon-γ release assay for the diagnosis of active tuberculosis among HIV-seropositive individuals: a systematic review and meta-analysis

**DOI:** 10.1186/s12879-016-1687-8

**Published:** 2016-07-22

**Authors:** Zhen-yu Huo, Li Peng

**Affiliations:** Department of Respiratory Medicine, the First Affiliated Hospital of Chongqing Medical University, No. 1, Youyi Rd, Chongqing, Yuzhong 400016 China

**Keywords:** Diagnostic accuracy, Interferon-γ release assay, Active tuberculosis, HIV-seropositive individuals, Systematic review

## Abstract

**Background:**

Although the interferon-γ release assay (IGRA) has become a widely accepted means for the diagnosis of latent tuberculosis infection (LTBI), the role of the IGRA in diagnosing active tuberculosis (ATB) among human immunodeficiency virus (HIV)-seropositive individuals remains controversial. Previous analyses did not set up rational inclusive criteria for screening articles with strict control groups and a gold standard for ATB diagnosis. Therefore, we conducted a systematic review of the latest evidence to evaluate the accuracy of IGRA for HIV-seropositive patients.

**Methods:**

Initially, we searched the EMBASE, Cochrane and MEDLINE databases to find research articles published from January 2000 to October 2015 that used the QuantiFERON-TB Gold In-Tube assay (QFT-IT) or the T-SPOT.TB assay (T-SPOT) to diagnose ATB among HIV-seropositive individuals. We separately calculated the pooled sensitivity, specificity, and proportion of indeterminate events and then summarized the results using forest plots to estimate the accuracy of the QFT-IT and T-SPOT assays.

**Results:**

A total of 1,743 studies were discovered after searching; 11 studies met our selection standards and were included for meta-analysis. The pooled sensitivity and specificity of the QFT-IT assay were 69 % (95 % CI, 50–84 %, I^2^ = 85.22 %) and 76 % (95 % CI, 53–90 %, I^2^ = 98.16 %), respectively, and the optimum area under the curve (AUC) was 0.78 (95 % CI, 0.74–0.82). The pooled sensitivity and specificity of the T-SPOT assay were 89 % (95 % CI, 66–97 %, I^2^ = 94.48 %) and 87 % (95 % CI, 38–99 %, I^2^ = 97.92 %), respectively, and the AUC was 0.93 (95 % CI, 0.90–0.95). The pooled ratios of the indeterminate results of the QFT-IT and T-SPOT assays were 0.07 (95 % CI, 0.06–0.09, I^2^ = 74.8 %) and 0.19 (95 % CI, 0.15–0.24, I^2^ = 88.3 %), respectively, calculated using the fixed effect model, and 0.08 (95 % CI, 0.06–0.12, I^2^ = 74.8 %) and 0.10 (95 % CI, 0.03–0.25, I^2^ = 88.3 %), respectively, calculated using the random effects model.

**Conclusions:**

The IGRA does not appear to be optimal for the clinical confirmation of ATB cases in HIV-seropositive patients; however, the T-SPOT assay may have greater accuracy in distinguishing ATB cases among HIV-infected individuals than the QFT-IT assay, while the QFT-IT assay appears to reduce the occurrence of indeterminate results. Furthermore, modification and additional trial designs are required to improve diagnostic effectiveness.

## Background

*Mycobacterium tuberculosis* infection has become one of the leading causes of mortality among patients living with acquired immunodeficiency syndrome (AIDS) caused by infection with the human immunodeficiency virus (HIV). In fact, one third of deaths among HIV-seropositive patients are due to tuberculosis (TB). The World Health Organization (WHO) reported 9.6 million TB cases worldwide in 2014 and 1.2 million HIV-seropositive TB cases. These cases were especially prominent in Africa, where HIV-seropositive TB cases represented 74 % of the total HIV/TB co-infections [1]. The high occurrence and mortality rates of TB among HIV-seropositive individuals urgently require a novel approach to practical TB diagnostics [2].

The gold standard for active tuberculosis (ATB) diagnosis remains bacteriological positive culture of *Mycobacterium tuberculosis* in sputum, bronchoalveolar lavage, and/or tissue biopsy; however, these methods are time-consuming [3, 4]. It may also be difficult to obtain an appropriate culture sample from HIV-seropositive individuals suffering from TB [5, 6]. Nevertheless, clinical and radiographic manifestations of ATB lack reliability among HIV-seropositive patients and tend to delay the diagnosis of ATB and initiation of appropriate anti-TB therapy [7, 8].

In the past 15 years, as an immunodiagnostic method, the interferon-γ (IFN-γ) release assay (IGRA) was developed to distinguish patients suffering from TB. The IGRA is a blood test that detects the immune response to *Mycobacterium tuberculosis*-specific antigens (early secreted antigenic target 6 kDa, or ESAT-6 and culture filtrate protein 10 kDa or CFP-10) in vitro. Currently, two commercial IGRAs are recommended and available for diagnosing TB in the clinic: an enzyme-linked immunospot assay [T-SPOT.TB assay (T-SPOT), Oxford Immunotec, Abingdon, UK] and an enzyme-linked immunosorbant assay [QuantiFERON-TB Gold In-Tube assay (QFT-IT), Cellestis, Carnegie, Australia] [9]. However, the limitations of the IGRA for practical applications in clinical work have prompted widespread controversy, particularly in immunocompromised population. Thus, it is essential to perform a comprehensive evaluation of the performance of the IGRA for diagnosing ATB among HIV-seropositive individuals based on latest evidence to guide clinicians.

We conducted a comprehensive systematic review and meta-analysis of the latest evidence to evaluate the accuracy of IGRA in diagnosing ATB among HIV-infected patients to guide the application of the IGRA in immunocompromised population.

## Methods

This systematic review and meta-analysis was conducted within the guidelines of the preferred reporting items for systematic reviews and meta-analyses (PRISMA) statement [10]. Because this research was a systematic review of published articles, ethical approval was not required. *Mycobacterium tuberculosis* culture was adopted as the gold standard for the diagnosis of ATB in this systematic review.

### Data sources and search strategy

We systematically searched all studies that estimated the accuracy of the IGRA (using two commercial IGRAs: QFT-IT and T-SPOT) for ATB among HIV-seropositive adult individuals. We searched the EMBASE, Cochrane and MEDLINE databases to find research articles published from January 1, 2000 to October 30, 2015. Searches were implemented using combinations of the following terms: “tuberculosis”, “active tuberculosis”, “interferon-gamma release assay”, “QFT-IT”, “T-SPOT”, “CFP-10”, “ESAT-6”, “IGRA”, “acquired immunodeficiency syndrome”, “human immunodeficiency virus” and “HIV”. We searched for additional references from review articles, guidelines and conferences when necessary.

### Study selection

In this systematic review, two investigators (ZY. Huo and L. Peng) independently completed a primary scan to screen references that were potentially appropriate based on the titles and abstracts. Studies that only evaluated the performance of IGRA for diagnosing active TB in HIV-seropositive patients on blood samples were included. Then, a secondary selection was completed by reviewing the full text of the article. Articles were excluded if one of the follow criteria was fulfilled: 1) the study did not set up a non-ATB control group in HIV-seropositive patients; 2) experiments were performed with an older generation of the IGRA method, did not use a commercial IGRA, or the cut-off values of the IGRA were not the same; 3) ATB cases were not confirmed by *Mycobacterium tuberculosis* culture; 4) the number of HIV-seropositive individuals was less than 10; 5) studies where screened individuals had received anti-TB therapy; 6) repeat research by the same author; 7) original data was not included; and 8) reports of prospective studies, conference abstracts, reviews, guidelines, letter and case reports. All citations were screened and verified independently by two investigators. Group discussion took place when the two investigators disagreed on the inclusive criteria of a citation.

### Data extraction and quality assessment

Data from all included research studies were extracted and crosschecked by two investigators independently. The following data from English-language articles were extracted: 1) author, publication year of article, calendar period of research, and the nation where the research was performed; 2) the age group of the enrolled participants; 3) the type of commercial IGRA studied; 4) the proportion of ATB among HIV-seropositive participants diagnosed by culture; and 5) the amount of indeterminate, negative and positive results for each group among the participants. The quality of all included studies was evaluated using the quality assessment of diagnostic accuracy studies-2 (QUADAS-2) checklist, which is a validated and widely used criterion for the diagnostic accuracy of trials [11].

### Statistical analysis

We adopted the standard methods recommended for systematic meta-analyses of diagnostic test evaluations [12, 13]. Statistical data analysis software programs (Stata/MP 13.1 and R for windows 3.2.2) were used for the meta-analysis. For each study, we separately calculated the sensitivity, specificity, and proportion of indeterminate events with the corresponding 95 % confidence intervals (95 % CI). Then, we summarized and synthesized these results using forest plots. Only cases with a confirmed *Mycobacterium tuberculosis* diagnosis by direct culture were classified as ATB patients, and the remaining cases were assumed to be non-ATB patients. Indeterminate results were excluded prior to calculating sensitivity and specificity if reported [14–16]. Statistically significant inconsistencies across articles were estimated by adopting the I^2^ statistic to examine the influence of authentic variability instead of errors associated with diagnostic sampling. The separated sensitivity and specificity of a single test for each study were used to generate a symmetric receiver operator characteristic (SROC) curve, which summarized the overall test performance and represented the variation occurring in the diagnostic accuracy among studies [17–19].

## Results

### Search results and study characteristics

**Fig. 1:**
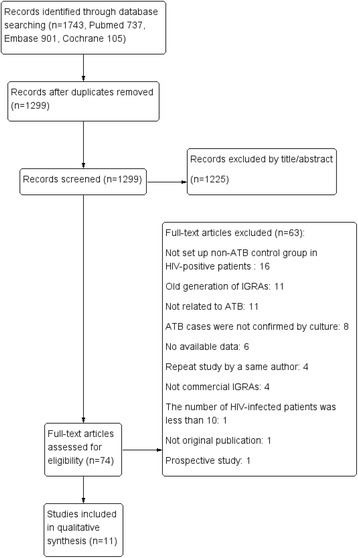
Flowchart diagram of the search strategy for study selection. IGRAs: Interferon-γ release assays; ATB: active tuberculosis

A total of 1,743 studies were identified after searching in the EMBASE, Cochrane and MEDLINE databases. After the full text of the articles was assessed for eligibility, 11 studies met our selection standards and were included in the meta-analysis [20–30]. Figure 1 summarizes the selection process for review articles with the detailed number of articles in each step. All articles were original publications and were published in English. The 11 selected studies included a total of 2,481 HIV-seropositive individuals with 305 culture-confirmed ATB cases. Table 1 shows the characteristics of the reviewed studies and the primary results. Nine studies estimated the performance of the QFT-IT assay, whereas four studies estimated the performance of the T-SPOT assay. Two studies reported the performance of both the QFT-IT and T-SPOT assays. Nine studies were performed in high-burden counties (more than 40 cases per 100 thousand population), whereas two studies were performed in low/intermediate-burden countries (less than 40 cases per 100 thousand population, specifically Austria and Italy) [31]. Almost all studies reported a median CD4+ T-lymphocyte count of less than 400 cells/mm^3^, except for three studies that did not report the median CD4+ counts in the text. Additional specific information with respect to the studies included in this systematic review is available upon request.

**Table 1:** General characteristics of the reviewed studies and the primary results

				Age (years)^b^		CD4+ counts (cells/μL)^c^			Assay results^e^				
Investigators	Year	Country	ATB/non-ATB patients enrolled^a^	ATB patients	Non-ATB patients	ATB patients	Non-ATB patients	IGRA method^d^	TP	FP	FN	TN	I
Aichelburg	2009	Austria	8/822	39 (32–47)	39 (32–47)	393 (264–566)	393 (264–566)	QFT-IT	7	37	1	738	47
Davarpanah	2009	Iran	11/165	38 (23–60)	38 (23–60)	360 (34–1300)	360 (34–1300)	QFT-IT	4	45	7	107	13
Veldsman	2009	South Africa	30/30	NR	NR	NR	NR	QFT-IT	9	11	15	16	9
Cattamanchi	2010	Uganda	112/100	33 (27–40)	33 (27–40)	49 (16–160)	49 (16–160)	T-SPOT	61	34	23	40	54
Legesse	2010	Ethiopia	6/21	34.2 (18–70)	34.2 (18–70)	NR	NR	QFT-IT	5	10	1	10	1
Leidl	2010	Uganda	19/109	33.4 ± 6	34.1 ± 8.3	182 (118)	283 (226)	T-SPOT	17	59	0	46	6
								QFT-IT	13	74	6	31	4
Tan	2010	Taiwan	9/30	39.6 (30–52)	39.1 (19–67)	112 (31–332)	145 (0–596)	T-SPOT	8	2	1	28	0
Bua	2011	Italy	9/64	41 (21–63)	41 (21–63)	270 (4–897)	270 (4–897)	QFT-IT	2	6	6	47	12
Rangaka	2011	South Africa	50/729	35 (31–40)	36 (31–42)	169 (98–239)	198 (136–315)	QFT-IT	32	298	15	383	51
Lagrange	2013	India	38/54	38.0 (30.5-42.0)	38.0 (30.5-42.0)	NR	NR	QFT-IT	26	19	3	29	15
Markova	2014	Bulgaria	13/52	43 (29 – 63)	37 (21–66)	195 (15–450)	248 (36–785)	T-SPOT	8	0	1	48	8
								QFT-IT	12	0	1	49	3

^a^All patients in the study were HIV-seropositive. ATB, active tuberculosis

^b^The values represent the means ± SD or medians with corresponding interquartile ranges (IQRs). NR, not reported in the study

^c^The values represent the means ± SD or medians with corresponding IQRs

^d^IGRA, interferon gamma release assay. QFT-IT, QuantiFERON-TB Gold In-Tube assay. T-SPOT, T-SPOT.TB assay

^e^ *TP* true positive, *FP* false positive, *FN* false negative, *TN* true negative, *I* indeterminate

### Quality of the selected studies

**Fig. 2:**
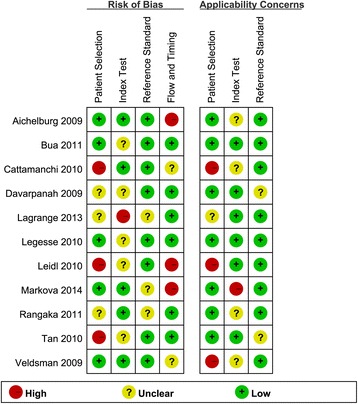
Methodological quality evaluation results of 11 studies sorted using the Quality Assessment of Diagnostic Accuracy Studies-2 (QUADAS-2) tool. See references [20–30] for details

Figure 2 shows the quality of the studies included using the QUADAS-2 tool. Of these 11 articles, 6 studies showed a high risk of bias, whereas 4 studies showed a high risk of applicability concerns. Additional information about the quality of the overall studies is shown in Fig. 2.

### Sensitivity and specificity of the QFT-IT

**Fig. 3:**
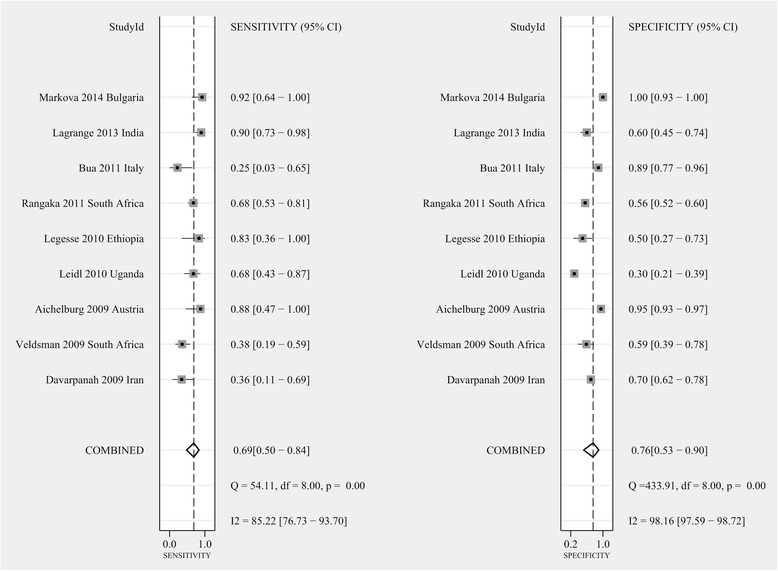
Forest plots of the pooled sensitivity and specificity of the QFT-IT assay for diagnosing ATB among HIV-seropositive individuals after excluding indeterminate results. See references [20–28] for details

**Fig. 4:**
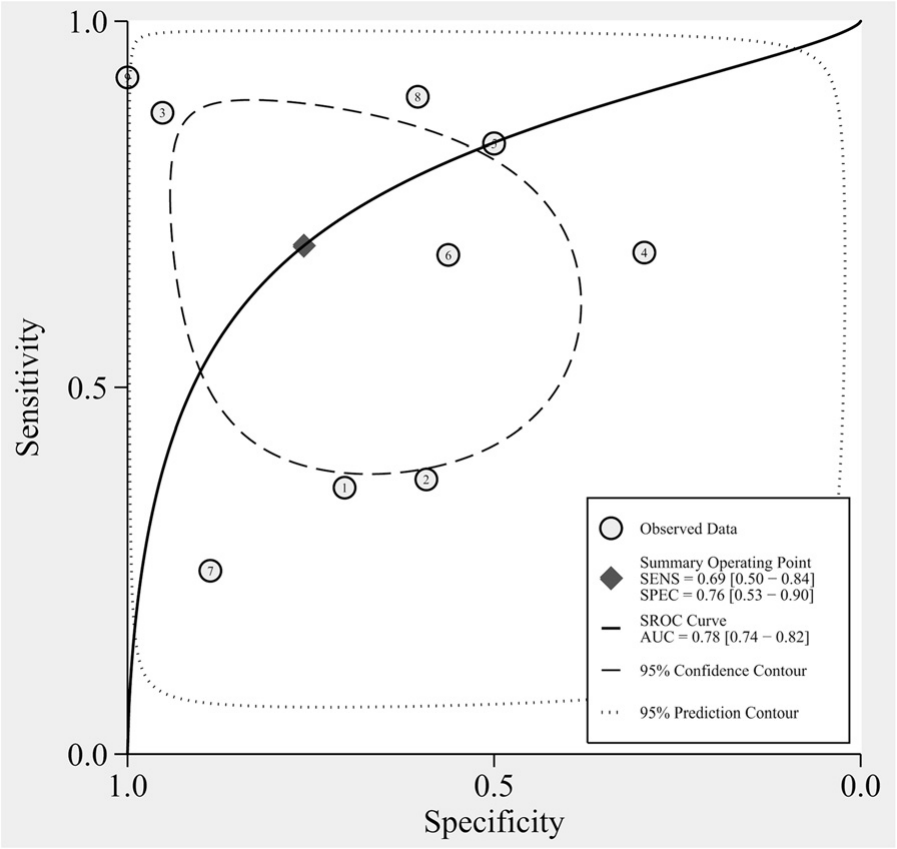
Symmetric receiver operator characteristic (SROC) curve for the QFT-IT assay. The SROC curve was derived by Stata/MP 13.1

After precluding the indeterminate QFT-IT events, 9 studies with a total of 2,230 participants were incorporated to calculate the combined sensitivity and specificity of the QFT-IT assays [20–28]. The pooled sensitivity of the QFT-IT assay for active culture-confirmed TB in HIV-seropositive individuals was 69 % (95 % CI: 50–84 %, I^2^ = 85.22 %), and the pooled specificity was 76 % (95 % CI: 53–90 %, I^2^ = 98.16 %, Fig. 3). We estimated the pooled diagnostic performance of the QFT-IT assay by generating SROC curves, which show the sensitivity versus the specificity, and by calculating the corresponding area under the SROC curves (AUC, Fig. 4). The SROC curve for QFT-IT was not located near the desirable upper left corner, and the optimum AUC was 0.78 (95 % CI, 0.74–0.82).

### Sensitivity and specificity of the T-SPOT

**Fig. 5:**
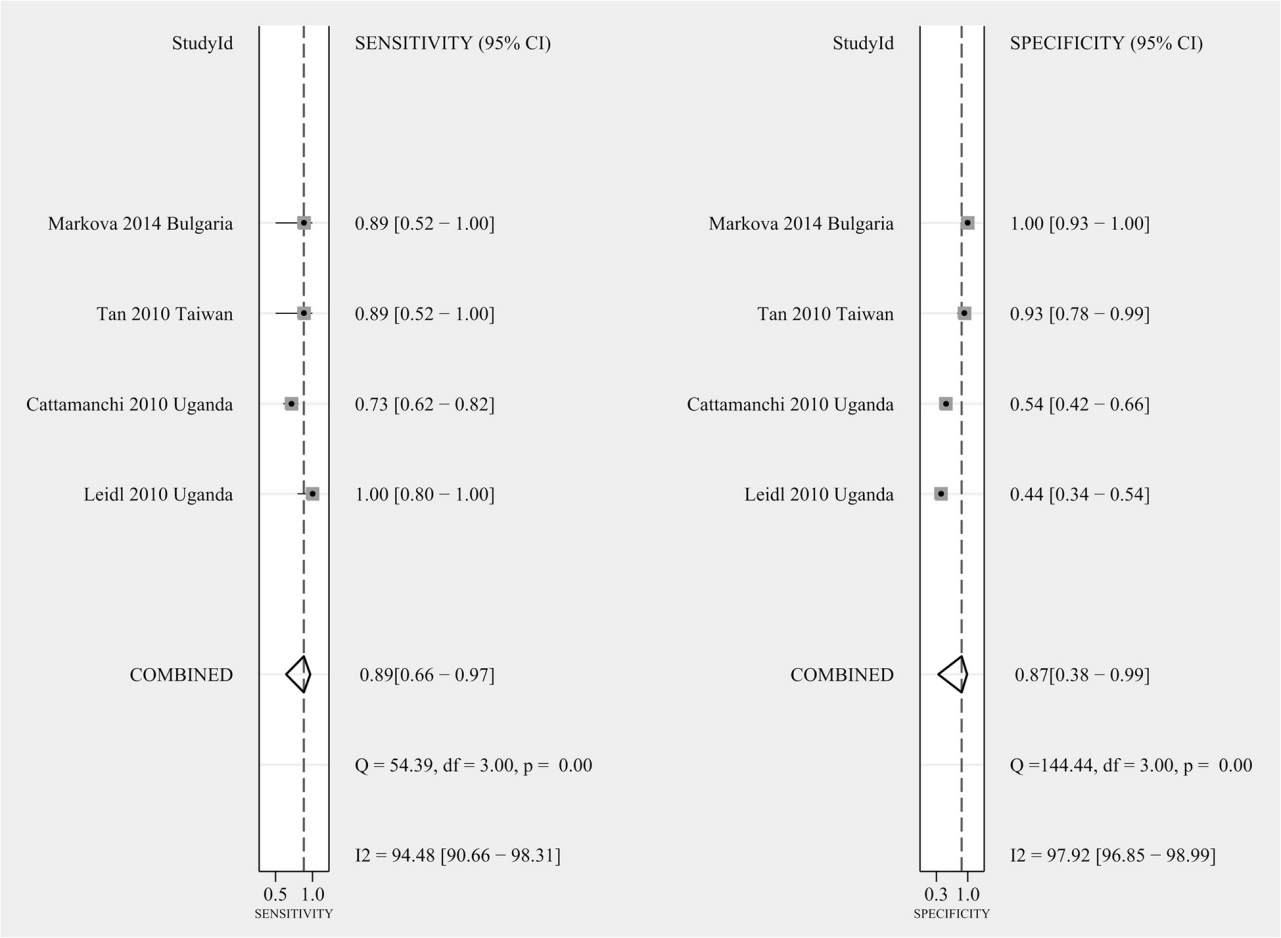
Forest plots of the pooled sensitivity and specificity of the T-SPOT assay for diagnosing ATB among HIV-seropositive individuals after excluding indeterminate results. See references [27–30] for details

**Fig. 6:**
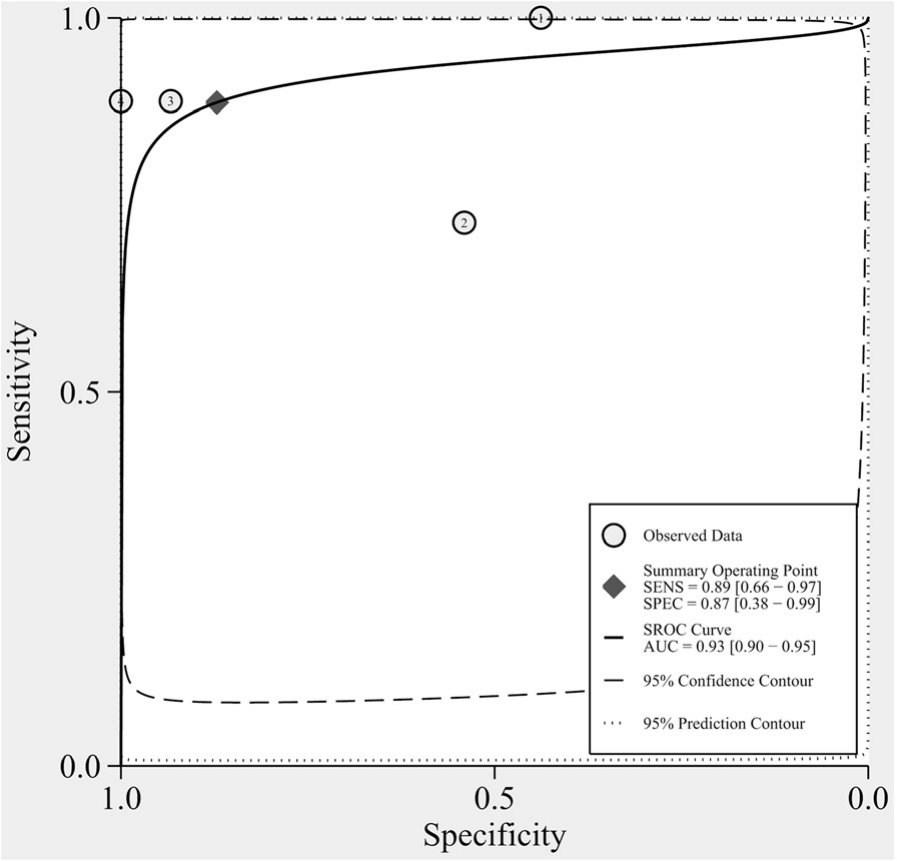
Symmetric receiver operator characteristic (SROC) curve for the T-SPOT assay. The SROC curve was derived by Stata/MP 13.1

At the same time, the combined sensitivity of the T-SPOT assay was calculated from four studies with a total of 444 individuals after excluding the indeterminate events [27–30]. The pooled sensitivity of the T-SPOT assay for active culture-confirmed TB was 89 % (95 % CI, 66–97 %, I^2^ = 94.48 %), whereas the pooled specificity of the T-SPOT assay was 87 % (95 % CI, 38–99 %, I^2^ = 97.92 %, Fig. 5). We calculated the SROC curve and the corresponding AUC for the T-SPOT assay using Stata/MP 13.1 (Fig. 6). Although the SROC curve for the T-SPOT assay was positioned in the desirable upper left corner, and the AUC was 0.93 (95 % CI, 0.90–0.95), these results may not represent the authentic performance of the T-SPOT assay, as evidenced by the high heterogeneity among the studies (I^2^ = 94.48 and 97.92 %, for sensitivity and specificity, respectively) and the small number of inclusive studies.

### Indeterminate events

**Fig. 7:**
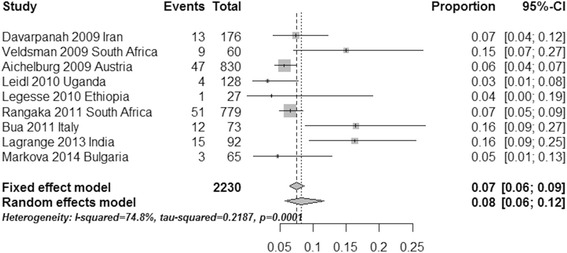
Forest plot of the pooled ratio of QFT-IT indeterminate events using the fixed effects model and the random effects model. See references [20–28] for details

**Fig. 8:**
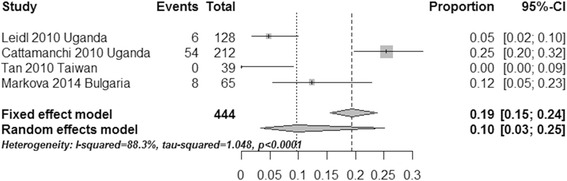
Forest plot of the pooled ratio of T-SPOT indeterminate events using the fixed effects model and the random effects model. See references [27–30] for details

To estimate the occurrence of indeterminate events using two commercial IGRAs, we calculated the pooled ratio of indeterminate results using a data analysis software program (R for Windows 3.2.2). The pooled ratio of QFT-IT indeterminate events was 0.07 (95 % CI, 0.06–0.09, I^2^ = 74.8 %) when using the fixed effect model; when using the random effects model, the pooled ratio of the QFT-IT assay was 0.08 (95 % CI, 0.06–0.12, I^2^ = 74.8 %, Fig. 7). When using the fixed effect model, the pooled ratio of T-SPOT indeterminate events was 0.19 (95 % CI, 0.15–0.24, I^2^ = 88.3 %); the pooled ratio for the T-SPOT assay was 0.10 (95 % CI, 0.03–0.25, I^2^ = 88.3 %) when using the random effects model (Fig. 8). Compared with the T-SPOT, the QFT-IT assay appeared to reduce the percentage of indeterminate results among HIV-seropositive individuals.

### Publication bias

**Fig. 9:**
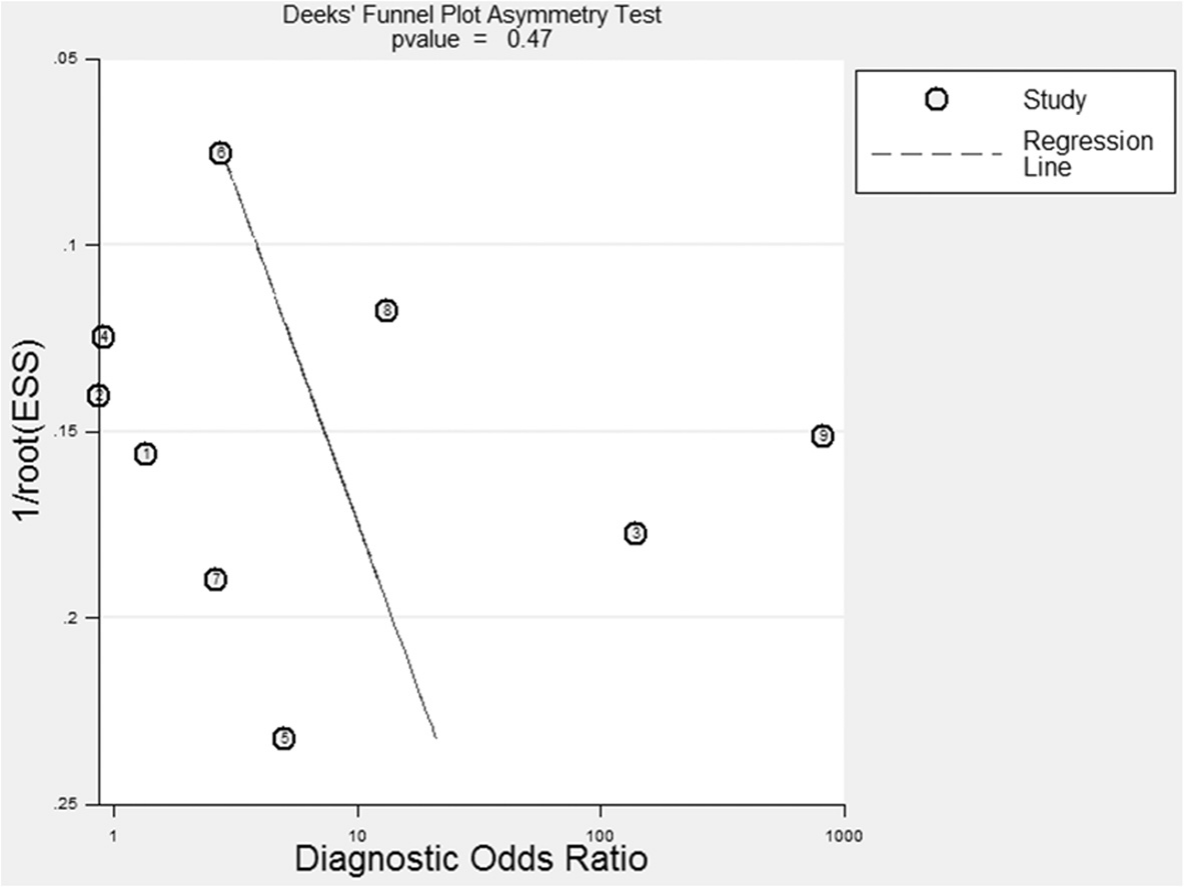
A Deeks’ funnel plot asymmetry test for evaluation of potential publication bias for the QFT-IT assay. This plot indicated a low risk of publication bias

**Fig. 10:**
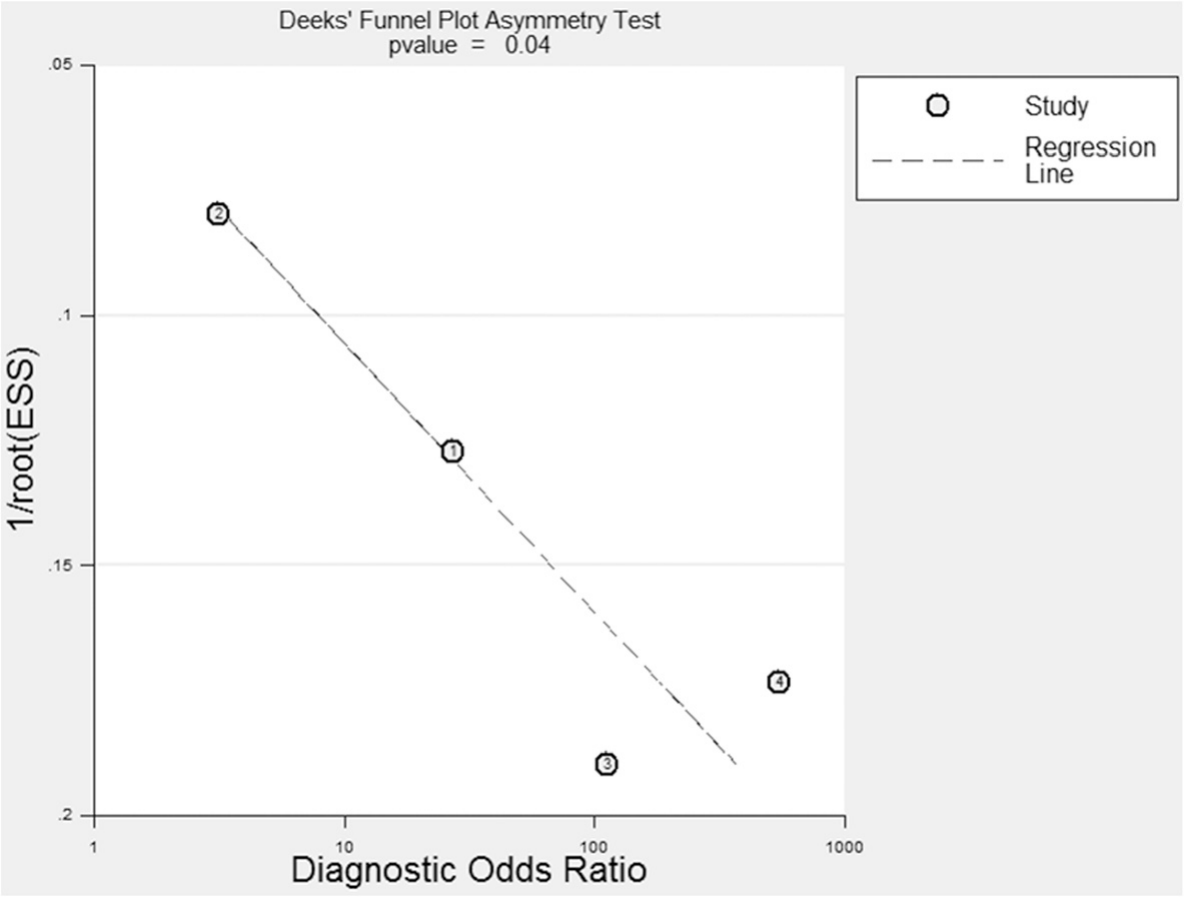
A Deeks’ funnel plot asymmetry test for evaluation of potential publication bias for the T-SPOT assay. This plot indicated a significant potential risk of publication bias

The publication bias of the 11 included studies was evaluated and analyzed using Deeks’ funnel plot asymmetry test (Figs. 9 and 10). Figure 9 indicates no significant asymmetry for the QFT-IT assay (*P* = 0.47), which suggested a low risk of publication bias for this assay. However, Fig. 10 shows an asymmetrical funnel plot for the T-SPOT assay (*P* = 0.04), which indicates a potential risk of publication bias.

## Discussion

The results of our systematic review, which differ from previous analyses, appear to suggest that the IGRA plays a limited role in diagnosing ATB in HIV-seropositive patients. However, this meta-analysis is an update and improves on previous articles in several respects [14, 32]. Compared to previous studies, we included the most recent publications prior to 2016 to perform a comprehensive and objective evaluation of the IGRA and set a more strict and rational inclusion criteria for this review. The search strategy of our study was broader, and a total of 1,743 studies were identified after comprehensively searching three databases. After screening using our precise inclusion criteria, only eleven studies met our selection standards and were included in the meta-analysis. Then, we used the latest validated quality assessment checklist tool, QUADAS-2, to evaluate the risk of bias and applicability concerns among the included studies in detail. We adopted the standard methods that are recommended for systematic meta-analyses and used two types of data analysis software programs to pool the statistics.

As shown in Fig. 2, using the QUADAS-2 tool, the quality of the 11 selected studies in this systematic review was relatively polytropical, with 6 studies showing a high risk of bias and 4 studies showing a high risk of applicability concerns. Only 4 studies exhibited a preferable quality and low risk of bias.

The pooled sensitivity and specificity of the QFT-IT assay were 69 and 76 %, respectively, for diagnosing ATB among HIV-seropositive patients after excluding indeterminate results, which were similar to the results of previous studies in which the sensitivity and specificity varied from 61–76.7 to 72–76.1 %, respectively [14, 32]. These experiments suggested that the QFT-IT assay was not sensitive and specific enough to select real ATB patients or to rule out people who are not suffering ATB among HIV-seropositive patients. When adopting the QFT-IT, greater than 30 % of ATB patients might be missed, and greater than 20 % of non-ATB patients might be diagnosed as having TB. The pooled sensitivity and specificity of the T-SPOT assay were as high as 89 and 87 %, respectively, indicating that the T-SPOT assay may have greater accuracy for distinguishing ATB cases among HIV-positive patients than the QFT-IT assay. These results were different from those of previous studies in which the sensitivity and specificity varied from 65–77.4 to 63.1–70 % [14, 32], respectively; this also showed that the T-SPOT assay may be more effective in this review. The differences in conclusions among this review and previous studies might be attributed to more reasonable inclusion criteria and latest references. Nevertheless, according to the small number of studies included and the high heterogeneity, the results calculated may not reflect the authentic accuracy of the T-SPOT in the diagnosis of ATB among HIV-seropositive patients. More experiments should be designed in the future to confirm the effectiveness of the T-SPOT assay.

The pooled ratios of the indeterminate QFT-IT and T-SPOT results were 0.07 and 0.19 when using the fixed effect model and 0.08 and 0.10 using the random effects model, respectively. Overall, the QFT-IT assay appeared to reduce the occurrence of indeterminate results among HIV-seropositive individuals. This conclusion seems more meaningful for diagnosis of LTBI, because an indeterminate result is the major limitation of the IGRA inimmunosuppressed subjects. The reason for the greater ratio of indeterminate results in the case of the T-SPOT might be associated with technical error due to the more sophisticated technical demand compared with the QFT-IT assay [32].

The SROC curve for the T-SPOT assay was positioned in the upper left corner, and the AUC was 0.93 (95 % CI, 0.90–0.95), which may suggest a more effective performance for distinguishing ATB cases among HIV-infected individuals than the QFT-IT assay, which produced an unsatisfactory AUC of 0.78 (95 % CI, 0.74–0.82). However, as evidenced by the high heterogeneity among the studies (I^2^ = 94.48 and 97.92 % for sensitivity and specificity) and the small number of included studies, the results may not accurately represent the performance of the T-SPOT assay. Furthermore, more additional trials are required to determine the efficiency of the T-SPOT assay.

The potential risk of publication bias was significant (*P* = 0.04) for the T-SPOT assay in our meta-analysis, as shown in Fig. 10. This indicated that preference may have been given to claims supporting the hypotheses in these publications when they were under review [27–30]. No significant asymmetry was observed for the QFT-IT assay (*P* = 0.47), which suggested a low risk of publication bias.

Our systematic review also has some limitations. Due to our strict selection and exclusion process, the number of included studies was only 11. Moreover, there was heterogeneity among the studies included in our analysis, and the difference between low/intermediate-burden countries and high-burden countries may be one of the reasons for the heterogeneity.

## Conclusions

In summary, the latest evidence provided in this systematic review and meta-analysis shows the current limited ability and accuracy of the IGRA for diagnosing ATB and ruling out non-ATB cases; therefore, the T-SPOT assay may be more accurate for distinguishing ATB cases among HIV-infected individuals, while the QFT-IT assay appears to reduce the occurrence of indeterminate results. Thus, the IGRA does not appear to be optimal for the clinical confirmation of ATB cases in HIV-seropositive individuals until its efficiency is verified, although modifications and additional trials may help to improve its effectiveness.

## Abbreviations

95 % CI, 95 % confidence intervals; AIDS, acquired immunodeficiency syndrome; ATB, active tuberculosis; HIV, human immunodeficiency virus; IFN-γ, interferon-γ; IGRA, interferon-γ release assay; IQRs, interquartile ranges; LTBI, latent tuberculosis infection; PRISMA, preferred reporting items for systematic reviews and meta-analyses; QFT-IT, quantiFERON-TB gold in-tube assay; QUADAS-2, quality assessment of diagnostic accuracy studies-2; SROC, symmetric receiver operator characteristic; TB, tuberculosis; T-SPOT, T-SPOT.TB assay; WHO, world health organization
